# Cultivation strategy optimization and pilot-scale production of *Spirulina subsalsa* grown in seawater and monosodium glutamate wastewater

**DOI:** 10.1186/s40643-025-00926-0

**Published:** 2025-07-31

**Authors:** Mingyan Liu, Liqun Jiang, Ze Yu, Meng Ma, Huiying Chen, Haiyan Pei

**Affiliations:** 1 School of Environmental Science and Engineering, Shandong University, Qingdao, 266237 China; 2 Department of Environmental Science and Engineering, Fudan University, Shanghai, 200433 China; 3 Shandong Provincial Engineering Center on Environmental Science and Technology, Jinan, 250061 China; 4 Institute of Eco-Chongming (IEC), Shanghai, 202162 China

**Keywords:** *Spirulina subsalsa*, Cultivation approach, Pilot-scale cultivation, Biomass composition

## Abstract

*Spirulina subsalsa* represents a promising candidate for commercial production. Yet, due to its unique pattern of attached growth followed by upward flotation, there is an urgent need to find a suitable cultivation strategy and achieve scale-up. In the low-cost medium of seawater plus monosodium glutamate wastewater, this study initially investigated the influence of aeration rate (0, 0.3, 0.6 L/min) and reactor geometry (*D*_*24*_*d*_*4.5*_, *D*_*18*_*d*_*8*_, *D*_*12*_*d*_*18*,_ *D*: diameter, cm; *d*: depth, cm) on microalgal growth, indicating that *Spirulina subsalsa* was suited for culturing in non-aerated, wide-shallow reactors. Then, in the single plastic reactor, diverse depths (2.25, 4.50, 9.00 cm) and surface areas (1000, 2000, and 3000 cm^2^) for culturing *Spirulina subsalsa* were optimised to 4.5 cm and 2000 cm^2^. Subsequently, a pilot-scale cultivation system with a volume of 162 L was constructed, adopting a semi-continuous culture mode. The average dry mass productivity was 0.12 g/L/d and 816.48 g of algal powder was harvested within 41 days. The mean contents of protein, carbohydrate and lipid were respectively 46.50%, 14.95% and 10.87%. Moreover, economic analysis demonstrated that the cost of produced algal powder was 8.30 USD/kg. In the future, *Spirulina subsalsa* has the potential to be developed into multiple products.

**Supplementary Information:**

The online version contains supplementary material available at 10.1186/s40643-025-00926-0.

## Introduction

Microalgae are promising biomass feedstocks, which have the potential to assist the carbon neutrality and energy transition goals (Kingsley et al. 2023). Particularly interesting is the Arthrospira (commercially known as Spirulina, hereinafter referred to as Spirulina) that is a helical cyanobacterium with high proliferation rate, strong environment adaptability and rich bioactive substances. Spirulina, one of the archaic organisms on Earth, absorbs carbon dioxide to produce oxygen through photosynthesis and first appeared about 3.6 billion years ago. Currently it has successfully become one of the algal species that have been cultivated on a large scale (Hidasi and Belay 2018).

In 1969, Sosa Texcoco Company established the first factory for producing Spirulina (Soni et al. 2017; Mehar et al. 2019). Since then, Spirulina manufacturers have proliferated. Commercial Spirulina occupies more than 30% of global microalgal biomass production market share, of which *Arthrospira platensis* and *Limnospira maxima* are two common species in large-scale production (Costa et al. 2019). Due to its superior biomass productivity, Zarrouk medium has become a common medium for cultivating these two species in recent years. As reported by Yuan et al. (2018), eight companies employed Zarrouk medium for culturing *Arthrospira platensis*, with the cost of the medium amounting to USD0.08 per litre. Nevertheless, due to the high expenditure involved, the reliance on chemical cultivation medium for commercial Spirulina production is unsustainable (Thevarajah et al. 2022).

Scientists have persistently investigated alternative nutrient sources or media to reduce cultivation cost, among which wastewater is a promising pathway (Cheah et al. 2016). The cost of producing *Golenkinia* SDEC-16 biomass was reduced by 30% by using wastewater and seawater, compared to BG11 medium (Yu et al. 2023). Numerous studies have shown that algal biomass cultured in wastewater-based medium is equivalent to or exceeds that in standard medium (Cheah et al. 2016; Shu et al. 2018). Several studies have been performed on Spirulina cultivation in diluted wastewater (Jiang et al. 2015, 2019, 2023). It was primarily found that monosodium glutamate wastewater (MSGW) — charactered as having high ammonia (around 50 g/L), high COD (around 200 g/L), and low pH (around 3) — can be diluted to cultivate *Spirulina subsalsa*. On this basis, a cultivation system based on seawater-supplemented MSGW was established (Jiang et al. 2019). The combination of wastewater and seawater for microalgal cultivation saves freshwater resource usage and lessens nutrient outlay (Zhou et al. 2024a, b).

Yet the ability to grow rapidly in this medium is intimately tied to selected species. *Spirulina subsalsa* was a more suitable strain, with a higher biomass than that of *Arthrospira platensis* in seawater plus MSGW medium. And *Spirulina subsalsa* had superior protein content (above 40%), phycocyanin content (7.5%) and purity (nearly 0.7) in 250 mL conical flasks (Jiang et al. 2015, 2019). Compared to the other two commercial Spirulina strains, *Spirulina subsalsa* has many advantages (Shiels et al. 2022; Szubert et al. 2018; Jiang et al. 2021), while the scale of *Spirulina subsalsa* production is relatively limited (Park et al. 2018). The key distinction lies in the fact that *Spirulina subsalsa* was attached growth followed by upward flotation. This unique growth characteristic imposes special requirements on the cultivation strategy of *Spirulina subsalsa*. Therefore, under low-cost cultivation conditions, it is crucial to seek a suitable cultivation approach for culturing *Spirulina subsalsa* on a large scale, thereby offering more alternatives for commercial Spirulina production.

Previous studies investigating *Spirulina subsalsa* in seawater supplemented MSGW mainly focused on algal biomass and product characteristics in conical flasks. For *Spirulina subsalsa* with the vertical migration trait, there is still limited information about the cultivation strategy and pilot cultivation in seawater-supplemented MSGW medium. Hence, this paper focuses on four aspects: (1) Suitable cultivation strategies for culturing *Spirulina subsalsa* were explored; (2) Depth and surface area for culturing *Spirulina subsalsa* in a single apparatus were optimised; (3) Using seawater supplemented with MSGW, a 162 L pilot-scale cultivation system was constructed and operated stably for six periods; (4) Economics and carbon emission were analysed, and proposals for the future were outlined.

## Materials and methods

### Microalgal strain and medium

*Spirulina subsalsa* was purchased from the Freshwater Algae Culture Collection of the Institute of Hydrobiology (Wuhan, China), and pre-cultured in sterile Spirulina medium (SP) that was recommended by this platform to culture the strain (Jiang et al. 2023). MSGW, a brown liquid produced through the stage of glutamic acid extraction, was collected from the Liangshan Linghua Gourmet Powder factory in Jining, China. In the process of separating sodium glutamate, a solution was produced that contained ammonium sulfate along with various other impurities. Then the solution underwent air float sterilization followed by the removal of ammonium sulfate. The wastewater, obtained by heating with high-temperature steam, was discharged from the equipment, which was the MSGW used in our experiment. MSGW was filtered by six layers of gauze, and seawater collected from Qingdao (China) was filtered by a 0.22 μm membrane.

The MSGW-seawater ratio was 1/1000 (V_MSGW_/V_seawater_) for culturing *Spirulina subsalsa*, established through preliminary trial. The total nitrogen (TN), total phosphorus (TP), ammonium nitrogen (NH_3_-N), dissolved organic carbon (DOC), suspended solids, turbidity, electrical conductivity, pH and salinity in the S + MSGW medium were 55.26, 3.53, 47.51, 120.01, 4.22 mg/L, 1.26 NTU, 39.37 mS/cm, 6.85 and 2.36%, respectively. *Spirulina subsalsa* was phototrophically grown with homogeneous illumination of 3000 lux provided by daylight fluorescent lamp tubes (Philips, 36 W). The temperature for culturing this algal strain was maintained at 25 °C in an artificial climate room.

### Experimental design 1: the exploration of diverse cultivation strategies

Initially, growth characteristics of *Spirulina subsalsa* were investigated under aerated and non-aerated cultivation conditions. Specifically, 2 L of culture medium was introduced to conical flasks, which were then sealed for non-aerated cultivation (*NA*). For the aerated groups, an equal volume of culture medium was added and an aeration device was installed, set at aeration rates of 0.3 (*A*_*0.3*_) and 0.6 (*A*_*0.6*_) L/min through the flowmeters, respectively.

Subsequently, cylindrical reactors with a cultivation volume of 2 L were employed for culturing *Spirulina subsalsa* under non-aerated conditions. With the cultivation volume kept constant, different combinations of diameter (*D*, cm) and depth (*d*, cm) were set to preliminarily explore a suitable reactor for microalgal cultivation. Three groups were established, namely *D*_*24*_*d*_*4.5*_, *D*_*18*_*d*_*8*_ and *D*_*12*_*d*_*18*_. In section “Experimental design 1”, initial inoculum for all groups was approximately 0.1 g/L and culture medium was S + MSGW, with top-lighting at an intensity of 3000 lux. Following a 3-day cultivation period, *Spirulina subsalsa* was harvested and then freeze-dried (FDU-1200, EYELA, Japan).

### Experimental design 2: the optimisation of depth and surface area

Based on the optimized cultivation strategy, single-variable refinement was carried out for the depth and surface area (*SA*, cm²) in a single apparatus. To facilitate manual handling, the surface shape of the apparatus was modified from orbicular to rectangular. A previous study demonstrated that light was able to just penetrate 5 cm of culture when the microalgal dry mass was 0.45 g/L (Raeisossadati et al. 2019). To guarantee effective light exposure, cultivation depth for this *Spirulina subsalsa* was optimized. Based on microalgal growth, diverse depths (*d*_*2.25*,_ *d*_*4.50*_, and *d*_*9.00*_) were designed with a length (*L*, cm) of 38 cm and width (*W*, cm) of 26 cm. To expand the scale with convenient manual operation, various surface areas (*SA*_*1000*_, *SA*_*2000*_, and *SA*_*3000*_) of length × width (*L*_*38*_*W*_*26*_, *L*_*71*_*W*_*28*_, and *L*_*93*_*W*_*43*_) were then investigated at the appropriate depth. Besides, the lamps were above the apparatus and incident light at the liquid surface was 3000 lux.

Microalgal inoculum was transferred to S + MSGW medium, in which initial biomass concentration remained approximately 0.1 g/L. A thin and transparent film was placed over the plastic apparatus to prevent water evaporation. This cultivation mode was semi-continuous cultivation, and the medium was reused to conserve resources. One experiment period was approximately 6 days: the initial cultivation period was 3 days and the majority of microalgal biomass was harvested using the scoop net, with retaining approximately 0.1 g/L of microalgal biomass in the medium. After supplementing 1/2000 MSGW, the experiment progressed to the subsequent phase. Microalgae were re-cultured for approximately 3 days and harvested once more. All harvested microalgae were subsequently washed with ultrapure water and then freeze-dried to obtain algal powder.

### Experimental design 3: pilot-scale cultivation

**Fig. 1:**
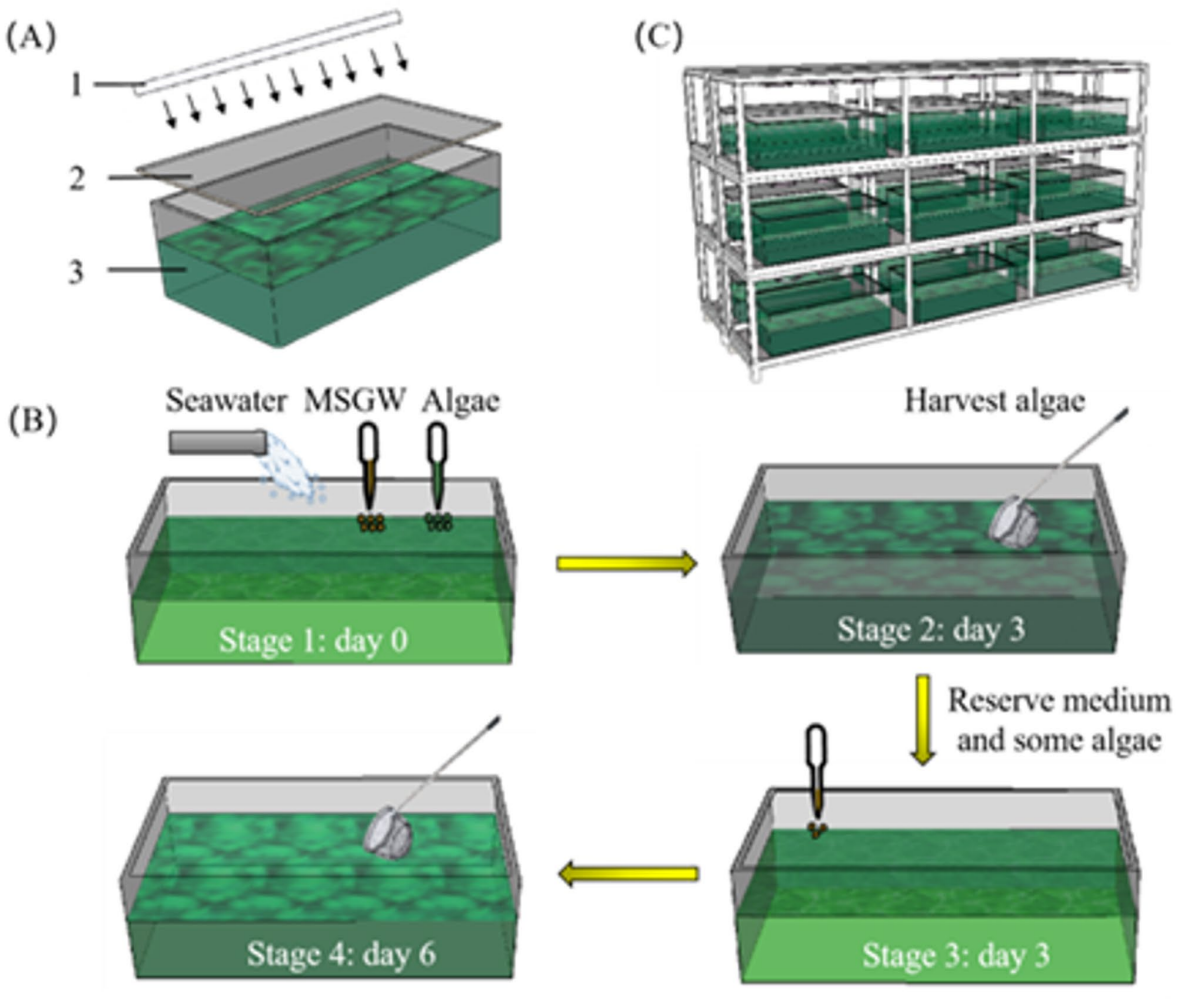
The culture system and process of cultivating *Spirulina subsalsa*. (**A**) Diagram of single vessel for culturing *Spirulina subsalsa*. 1: daylight fluorescent lamp tube. 2: film for waterproof evaporation. 3: single cultivation vessel. (**B**) Cultivation process for *Spirulina subsalsa*. (**C**) Overall diagram of the culture system

The culture system of *Spirulina subsalsa* was illustrated (Fig. 1). To save floor space, three hierarchically arranged shelves were constructed. Lamps were positioned above each layer to ensure a constant illumination of 3000 lux (24 h/d). Eighteen apparatuses with suitable dimensions were placed separately on the shelves, thereby facilitating an expansion of the cultivation scale. The total volume for culturing *Spirulina subsalsa* was 162 L, with an effective volume of 9 L in each apparatus. To validate stable operation, the overall cultivation period was specified as 41 days, comprising six periods. All experiments on *Spirulina subsalsa* involved performing three parallel experiments independently, and the data are reported as the mean ± standard deviation.

### Microalgal growth and morphology

*Spirulina subsalsa* growth was indicated by measuring mass on the harvest day due to uneven distribution of filamentous cyanobacteria cells. For observing morphological changes of microalgal cells, an optical microscope (CX31, Olympus, Japan) was employed. The indices of dry mass (*DM*, g/L), biomass productivity (*P*_*b*_, g/L/d) and specific growth rate (*µ*, d^-1^) are employed to reflect the content of microalgal weight in the culture medium, the efficiency of biomass accumulation, and the microalgal growth rate per unit time (Yu et al. 2023). The related formulae for biomass productivity (*P*_*b*_, g/L/d) and specific growth rate (*µ*, d^-1^) are as follows:

$$\:\begin{array}{c}{P}_{\text{b}}=\frac{{DM}_{2}-{DM}_{1}\:}{{t}_{2}-\:{t}_{1}}\#\left(1\right)\end{array}$$

$$\:\begin{array}{c}\mu\:=\frac{\text{ln}\left({DM}_{2}{/DM}_{1}\right)}{{t}_{2}\:-\:{t}_{1}}\#\left(2\right)\end{array}$$

where *DM*_1_ and *DM*_2_ (g/L) are the dry masses at times *t*_1_ and *t*_2_ (d).

### Nutrient levels

The TN, TP and NH_3_-N in S + MSGW medium were measured by an alkaline potassium persulfate digestion UV spectrophotometric method (HJ 636–2012), an ammonium molybdate spectrophotometric method (GB 11893-89), and by Nessler’s reagent spectrophotometry (HJ 535–2009), respectively. Dissolved organic carbon (DOC) was determined by TOC analyser (TOC-L CPN CN200, Shimadzu, Japan). Extracellular organic matter in S + MSGW medium was quantified by excitation–emission matrix (EEM) spectroscopy; the range of excitation/emission wavelengths, the interval of excitation/emission wavelengths and the scanning speed were respectively 220–450/250–550 nm, 5/1 nm, and 2400 nm/min. The average removal coefficients of nitrogen, phosphorus and carbon (N-ARC, P-ARC or C-ARC, mg/g) are presented to reflect the amount of nitrogen, phosphorus and carbon utilization per gram of microalgal biomass (Ma et al. 2023).

$$\:\begin{array}{c}N-,\:P-,\:or\:C-ARC=\frac{{C}_{2}\:-{C}_{1}}{DM}\#\left(3\right)\end{array}$$

where *C*_1_, *C*_2_ (mg/L) are concentrations of TN, TP or DOC on the initial and final days.

### The analysis of algal products

The lipid, carbohydrate, and protein were respectively determined using the chloroform–methanol method, the anthrone–vitriol method, and the sulfuric acid digestion method (Yu et al. 2023). The productivities of lipid, protein, and carbohydrate (*P*_L_, *P*_P_ and *P*_C_, mg/L/d) were found as follows:

$$\:\begin{array}{c}{P}_{L},{P}_{P},{P}_{C}=\frac{{C}_{\text{i}}\:}{T}DM\#\left(4\right)\end{array}$$

where *C*_*i*_ (%) is the content of lipid, protein or carbohydrate (as appropriate), and *T* (d) represents the overall cultivation time.

### Light attenuation

The light attenuation of diverse depths was described as Cornet model. The incident light intensity at the liquid surface was 3000 lux, and the emitted light intensity was measured using illuminance meter (1330; TES, China). The parameters of absorption coefficient (*E*_a_, m^2^/g) and scattering coefficient (*E*_s_, m^2^/g) in the following formula 5–7 were fitted by Origin 8.0 software. The basic equations are as follows:

$$\:\begin{array}{c}\frac{E}{{E}_{0}}\:\:=\frac{4{{\upalpha\:}}_{1}}{{\left(1+{{\upalpha\:}}_{1}\right)}^{2}{\text{e}}^{{{\upalpha\:}}_{2}}-{\left(1-{{\upalpha\:}}_{1}\right)}^{2}{\text{e}}^{{-{\upalpha\:}}_{2}}}\#\left(5\right)\end{array}$$

$$\:\begin{array}{c}{{\upalpha\:}}_{1}=\sqrt{\frac{{E}_{\alpha\:}}{{E}_{\alpha\:}+{E}_{S}}}\#\left(6\right)\end{array}$$

$$\:\begin{array}{c}{{\upalpha\:}}_{2}={DM\times\:L\times\:{\upalpha\:}}_{1}\times\:({E}_{{\upalpha\:}}+{E}_{S})\#\left(7\right)\end{array}$$

where *E* and *E*_*0*_ (lux) are emitted and incident light intensities; *L* (m) is light path.

### Economic analysis and carbon emission reduction

In accordance with present data for algal production, cultivation scale and time were expanded to 1 m^3^ and one year for economic analysis. The boundary of this production system encompassed the period of algal cultivation, harvesting, and drying, and two scenarios were set including fluorescent and natural light for algal illumination. The inputs covered raw materials, CO_2_ and electricity, while the output predominantly comprised algal powder. Carbon dioxide bio-fixation rate (*CDBR*, g/L/d) from the atmosphere was calculated by determining the carbon proportion in algal powder.

$$\:\begin{array}{c}CDBR={C}_{\text{c}\text{a}}{P}_{\text{b}}\frac{{M}_{cd}}{{M}_{c}}\#\left(8\right)\end{array}$$

where *C*_*ca.*_ (%) represents the proportion of carbon in algal powder, *M*_*cd*_ and *M*_*c*_ (g/mol) are the molecular mass of carbon dioxide and carbon.

## Results and discussion

### Optimization of the cultivation conditions

#### Algal feature of vertical migration

Photographs of *Spirulina subsalsa* exhibiting the trait of vertical migration were presented in Figure S1. Following the inoculation of *Spirulina subsalsa* into SP medium, the algal cells initially sank to the bottom under the influence of gravitational forces. Subsequently, the cells were observed to float off the bottom and ascend to the water surface. Remaining algal cells at the bottom continued to grow, eventually rising to the water surface. The process of reproduction and subsequent float was repeated for algal cells in the bottom. It is evident that *Spirulina subsalsa* was able to migrate vertically through buoyancy regulation.

The process of buoyancy regulation in cyanobacteria is typically dependent on the formation and subsequent degradation of gas vesicles within the cells, as well as the equilibrium between the synthesis and degradation of cellular ballast (Qi et al. 2023). Additionally, some gas bubbles generated through photosynthesis were attached to the surface of algae, significantly promoting the elevation of algal cells from the bottom (Figure S1). Light accessibility, crucial for sustaining photosynthesis, is inherently influenced by water depth (Raeisossadati et al. 2019). Based on these features, our initial objective was to ascertain whether aeration-induced mixing of *Spirulina subsalsa* would enhance the microalgal biomass. Subsequently, an initial assessment was conducted to determine the appropriate reactor dimensions for microalgal growth. Then, cultivation depth was optimized to guarantee an optimized growth environment, and this study carried out surface area optimization with a view to amplifying production output.

#### The exploration of cultivation strategy for Spirulina subsalsa

**Fig. 2:**
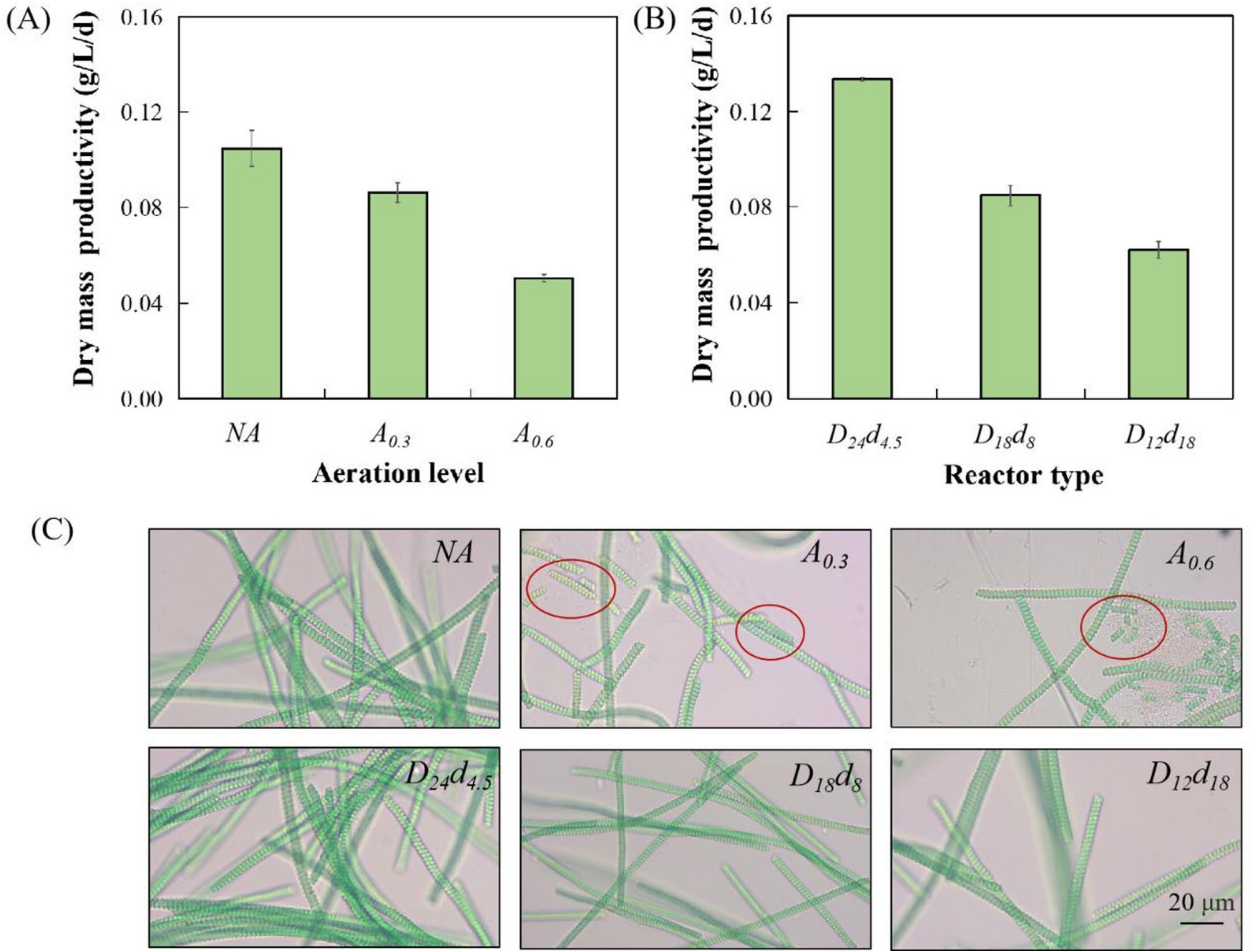
The cultivation mode optimization of *Spirulina subsalsa*. (**A**) The dry mass productivity in non-aeration (*NA*), aeration with the aeration rates of 0.3 (*A*_*0.3*_) and 0.6 (*A*_*0.6*_) L/min. (**B**) The dry mass productivity with diverse diameters (*D*, cm) and depths (*d*, cm; *D*_*24*_*d*_*4.5*_, *D*_*18*_*d*_*8*_ and *D*_*12*_*d*_*18*_) in non-aeration conditions. (**C**) Micrographs of *Spirulina subsalsa* grown in diverse modes on the harvested day (The red cycle means fragments of algal cells)

The growth characteristics of *Spirulina subsalsa* under different cultivation strategies were illustrated in Fig. 2 (A) and Figure S2 (A). Compared to the aerated group, the highest biomass, reaching up to 0.41 g/L, was observed without aeration. As aeration rate increased, there was a concomitant decrease in microalgal biomass. When aeration rates were 0.6 and 0.3 L/min, microalgal biomass respectively decreased by 29.93% and 13.42% in comparison to *NA* group. Majority of *Spirulina subsalsa* cells tend to accumulate at the water surface, while aeration resulted in a vertical redistribution of microalgae. The modification of their growth environment exerted an adverse impact on their growth. In addition, the thin cell wall of *Spirulina subsalsa* might be vulnerable to shear damage from aeration (López-Rosales et al. 2017; Zhao et al. 2023). Micrographs revealed that algal cells in the aerated group were shorter and fragmented, whereas those in the non-aerated group were longer and clustered. These findings indicated that for this strain of *Spirulina subsalsa*, non-aerated condition was an optimal approach.

The growth of *Spirulina subsalsa* at diverse diameters and depths without aeration was exhibited in Fig. 2 (B) and Figure S2 (B). The highest biomass of *Spirulina subsalsa* (0.50 g/L) was observed in *D*_*24*_*d*_*4.5*_ group. Conversely, the lowest biomass was 0.29 g/L, occurring in *D*_*12*_*d*_*18*_ group. The above data indicated an increase in microalgal growth when the depth was less than the diameter. It was possible that excessive cultivation depth impeded light penetration, thereby hindering microalgae growth. *Spirulina subsalsa* primarily floated at the water surface, where algal growth potential was constrained by their limited surface area (Nayana et al. 2023). Based on the above, this strain of *Spirulina subsalsa* was suitable to a wide-shallow reactor that the diameter exceeded the depth, under non-aerated conditions.

#### The depth and surface area optimization for culturing Spirulina subsalsa

**Fig. 3:**
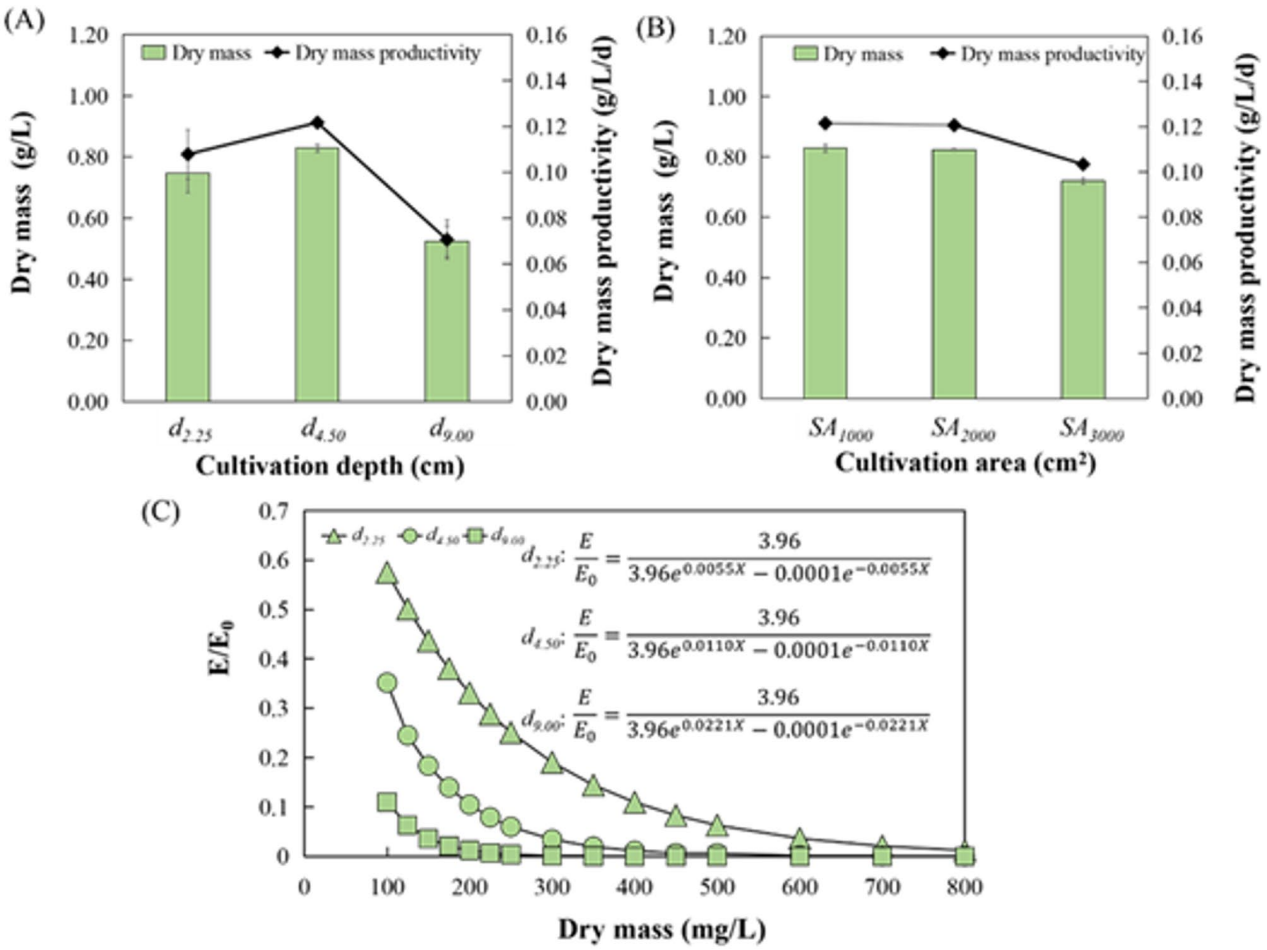
The optimization of depths (*d*_*2.25*,_ *d*_*4.50*_, and *d*_*9.00*_) and surface areas (*SA*_*1000*_, *SA*_*2000*_, and *SA*_*3000*_) in single apparatus for culturing *Spirulina subsalsa.* (**A**) The dry mass of diverse depths. (**B**) The dry mass of diverse cultivation areas. (**C**) The fitted curve of E/E_0_ at diverse depths according to the cornet model

Microalgal growth at different depths (*d*_*2.25*,_ *d*_*4.50*_, and *d*_*9.00*_) in the incubator was investigated (Fig. 3 (A). The dry mass content and productivity of *Spirulina subsalsa* were 0.84 g/L and 0.12 g/L/d in *d*_*4.50*_ group, representing the optimal performance compared with other depths. Besides, the dry mass content and productivity were 0.75 g/L, 0.11 g/L/d in *d*_*2.25*_ group and 0.52 g/L, 0.07 g/L/d in *d*_*9.00*_ group. The main reason for restricting microalgal growth at a depth of 9 cm is that deep depth affected the light utilization and further hindered microalgal photosynthesis according to the fitted curve of Fig. 3 (C). Hence, generation of gas bubbles through photosynthesis was affected, making algae at the bottom difficult to float and re-grow. At a relatively low depth, dry mass in that medium was reduced because algae were packed densely and lacked space to move around (Nayana et al. 2023).

At the optimized depth of 4.5 cm, microalgal growth in different surface areas (*SA*_*1000*_, *SA*_*2000*_, and *SA*_*3000*_) was explored for expanding production. Dry mass contents in the surface area of 1000, 2000, and 3000 cm^2^ were respectively 0.83, 0.82, and 0.72 g/L. The dry mass productivities in three systems all exceeded 0.1 g/L/d, which indicated robust growth performance. Surprisingly there was minimal variation in algal dry mass between *SA*_*1000*_ and *SA*_*2000*_, yet dry mass for *SA*_*3000*_ was lower than that of *SA*_*1000*_ and *SA*_*2000*_. Scaling up algal apparatus is a complex task because the control of environmental conditions has become complex and difficult, thus affecting Spirulina growth (Costa et al. 2019). *Spirulina subsalsa* initially accumulated at the bottom when they were introduced, and the interactions between the algae might change as the scale expands, potentially leading to a decrease in *SA*_*3000*_ biomass. For instance, Tan et al. (2019) pointed out that algae secrete alkaloids, fatty acids, or peptides, which inhibit their own growth. To maximize the productivity of algal powder, the surface area of 2000 cm^2^ was chosen for pilot-scale cultivation of *Spirulina subsalsa.* Herein optimized surface area and depth of 2000 cm^2^ and 4.5 cm were suitable for culturing *Spirulina subsalsa*.

### The pilot-scale cultivation of *Spirulina subsalsa*

#### The microalgal growth

**Fig. 4:**
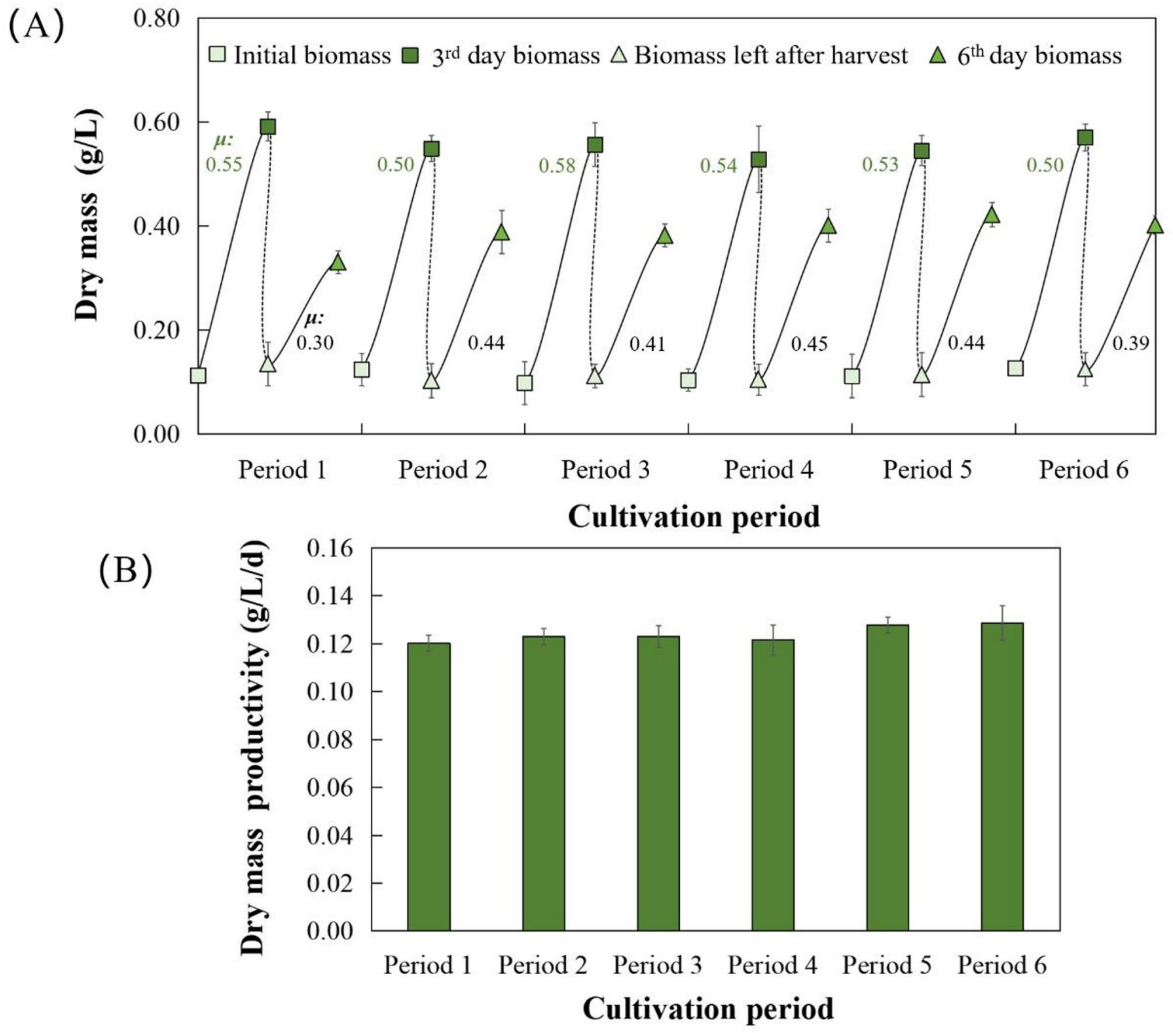
The pilot-scale cultivation of *Spirulina subsalsa*. (**A**) The growth data of *Spirulina subsalsa* in six periods ($\:\mu\:$ printed in green or black means specific growth rate in the first three days or the last three days, respectively). (**B**) The dry mass productivity in six periods

The dry mass content of *Spirulina subsalsa* was investigated to reflect microalgal growth (Fig. 4). The data from six growth periods indicated that pilot-scale cultivation of *Spirulina subsalsa* was stable in terms of biomass production. *Spirulina subsalsa* exhibited high dry mass content and productivity, reaching 0.84 g/L and 0.12 g/L/d, proving that it acclimatized remarkably well in seawater-based medium (Fig. 4; Table 1). For Spirulina cultivation, dry mass productivity (0.12 g/L/d) is higher than 0.05 g/L/d in modified Zarrouk medium and 0.07 g/L/d in swine wastewater (Mehar et al. 2019; Lu et al. 2020). Besides, mean *µ* reached 0.53 d^− 1^ for initial three days and 0.40 d^− 1^ for final three days, which were higher than other studies (Table 1). For a volume of 162 L, overall dry mass and productivity were 136.08 g and 19.44 g/d, respectively. After six cycles, 816.48 g of algal powder was produced for subsequent application.

**Table 1:** Comparison of microalgal growth and their metabolites with other studies

Algal species	Culture medium/ Reactor/Scale	*P*_b_ (g/L/d)	C_c,_ C_*p*_, C_L_ (%)	*P*_C_, *P*_*P*_, *P*_L_ (/mg/L/d)	Possible products	Cost analysis^a^	Reference
*Spirulina subsalsa*	S + 0.1%MSGW/Flat plate reactor/162 L	0.12	10.87/46.50/ 14.95	15.21/65.11/ 20.94	C-PC/ High-protein products	8.30 USD/kg	This study
*Spirulina* sp.	Essential nutrients/ Raceway pond/---	---	15.00/60.00/ 5.20	---	Spirulina food product	13.2–17.3 €/kg	Kingsley et al. 2023
*A. platensis*	Modified Zarrouk medium/ Raceway pond /1400 L	0.05	6.90/52.00/ 13.00	---	---	---	Mehar et al. 2019
Spirulina sp. LEB 18	Zarrouk medium/ Photobioreactor/1.8 L	0.05	19.3/66.6/6.1	12.74/43.95/ 4.03	C-PC/ High-protein products	---	Cordeiro et al. 2021
*Arthrospira* sp.	Essential nutrients/---/---	---	16.93/49.48/ ---	---	C-PC/Lipid/ Polysaccharides	10.84 USD/kg	Chaiklahan et al. 2018
*Spirulina* sp. LEB 18	Seawater-based medium/ Photobioreactor/1.5 L	0.14	10.00/30.90/42.40	58.80/---/---	---	---	Bezerra et al. 2020
C. *pyrenoidosa* SDEC-35	S + 0.4% MSGR/ Photobioreactor/25 L	0.06	13.74/26.79/ 40.9	9.72/14.39/ 25.90	Biodisel/ High-protein feed	---	Ma et al. 2024
*Scenedesmus armatus*	---	0.09	42.5/37.8/ 19.8	30.67/38.34/ 22.24	---	---	Ritcharoen et al. 2016
*Chlamydomonas reinhardtii*	TAP/Photobioreactor/2 L	0.12	---	26.09/---/ 21.34	Bioethanol/Pigment	---	Banerjee et al. 2021

^a^ means the cost analysis for producing 1 kg algal powder

The filaments of *Spirulina subsalsa* exhibited a tendency to disperse and expand gradually in the S + MSGW medium (Figure S1). In general, microalgae adjust their morphology — such as changes in cell size, cell shape or flagellar shedding — to resist adverse conditions. The morphology of *Spirulina subsalsa* did not change in the process of growth, which indicated high tolerance for seawater salinity. Previous studies have proved that the elongation and desaturation of fatty acids, the accumulation of lysine and methionine, and the secretion of sodium enables *Spirulina subsalsa* to adapt to the salinity in seawater-based medium, which is one of the reasons for its fast growth (Jiang et al. 2021; Chaiklahan et al. 2018; Lee et al. 2010). Based on the above, pilot cultivation of *Spirulina subsalsa* with the trait of vertical migration has been successfully achieved, and the production of algal powder can be stabilised.

#### The nutrient assimilation of Spirulina subsalsa

**Fig. 5:**
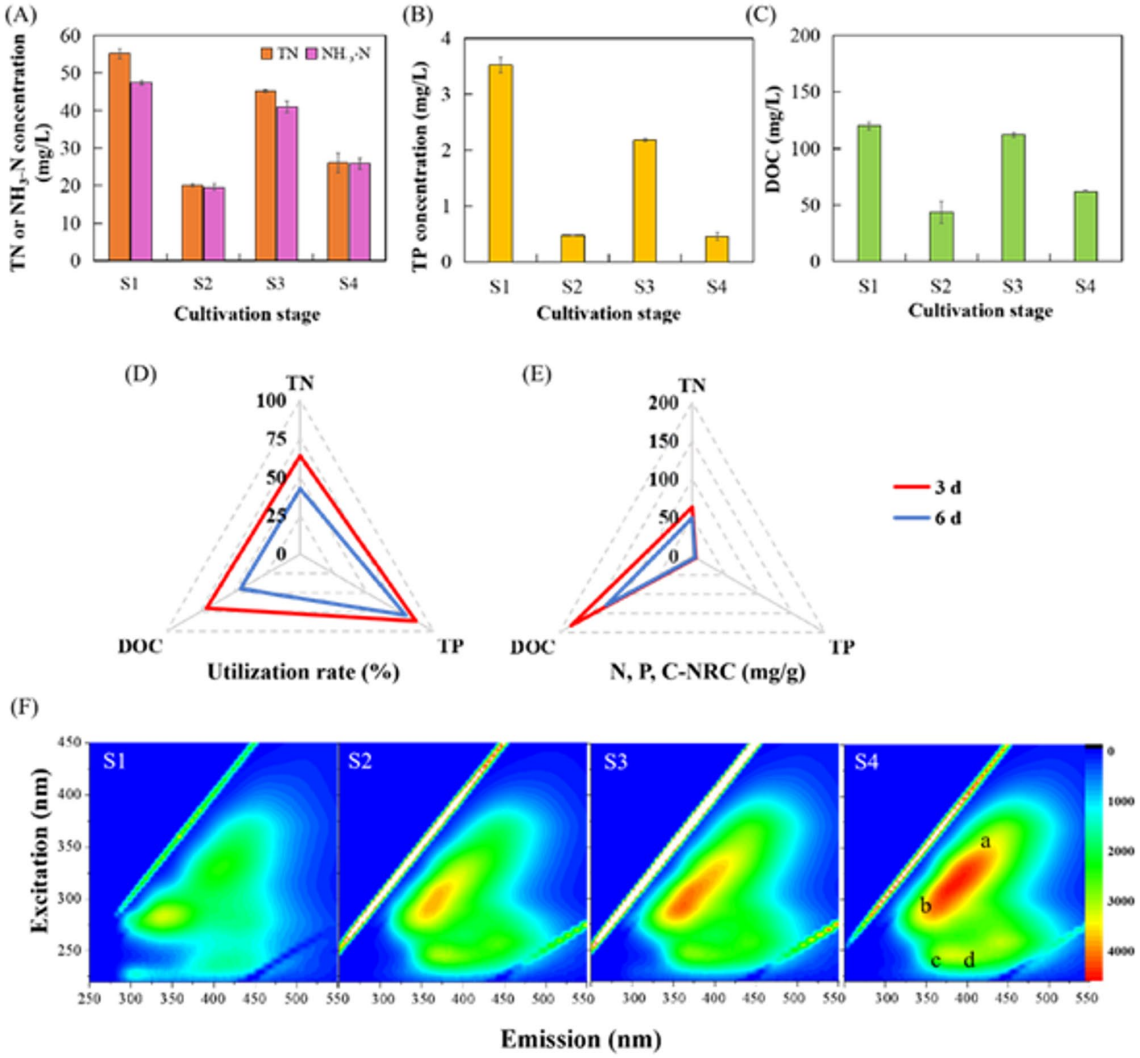
The nutrient utilization of *Spirulina subsalsa* in S + MSGW. (**A**) TN and NH_3_-N concentrations. (**B**) TP concentration. (**C**) DOC concentration. (**D**) The utilization rate of TN, TP and DOC. (**E**) The N-, P-, and C-NRC. (**F**) The fluorescence characteristics of excitation–emission matrix spectroscopy. S1, S2 mean the medium from the initial day and the 3^rd^ day, before adding a nutrient source. S3 and S4 respectively mean the medium of the 3^rd^ day after adding a nutrient source, and the 6^th^ day

The nutrient levels in S + MSGW medium were investigated (Fig. 5). Average N-AYC of *Spirulina subsalsa* was 56.33 mg/g, reducing from 55.26 mg/L to 20.02 mg/L in the first three days and from 45.16 mg/L to 26.03 mg/L in the last three days. NH_3_-N was the prevailing form of TN, making up 85.99% TN, which was absorbed at a value of 27.98 mg/L in the first three days and 15.20 mg/L in the last three days. It was demonstrated that NH_3_-N is directly utilized by microalgal cells and then forms amino acids through amination reaction with keto acids (Bezerra et al. 2020; Yang et al. 2016). When NH_3_-N was applied as a nitrogen source, high concentration of NH_3_-N would generate a toxic effect for microalgal cells. Based on microalgal morphology and growth characteristics, *Spirulina subsalsa* was able to tolerate this concentration of NH_3_-N and grew well in this experiment. Regarding TP, average P-AYC of *Spirulina subsalsa* was 4.97 mg/g, and its removal efficiencies was 86.74% over the first three days and 79.10% across the last three days.

As mentioned above, nutrients in the effluent can meet the nutritional needs of the microalgae and thus achieve efficient growth. However, growth rate of microalgae in the last three days of a period decreased compared to the first three days. According to EEM’s detection of Fig. 5 (F), the fluorescence of sections a, b, c, d all increased, meaning that humic-like acids (Ex/Em: 250–450 nm/380–550 nm), soluble microbial byproduct-like material (Ex/Em: 250–450 nm/280–380 nm), aromatic proteins (Ex/Em: 220–250/330–380 nm), and fulvic acid-like materials (Ex/Em: 220–250/380–550 nm) were more abundant in the medium on the 6^th^ day compared to that on the initial day. Extracellular organic matter produced in the culture medium may be one of the main factors that negatively influence algal growth in the last three days.

#### The products of Spirulina subsalsa

**Fig. 6:**
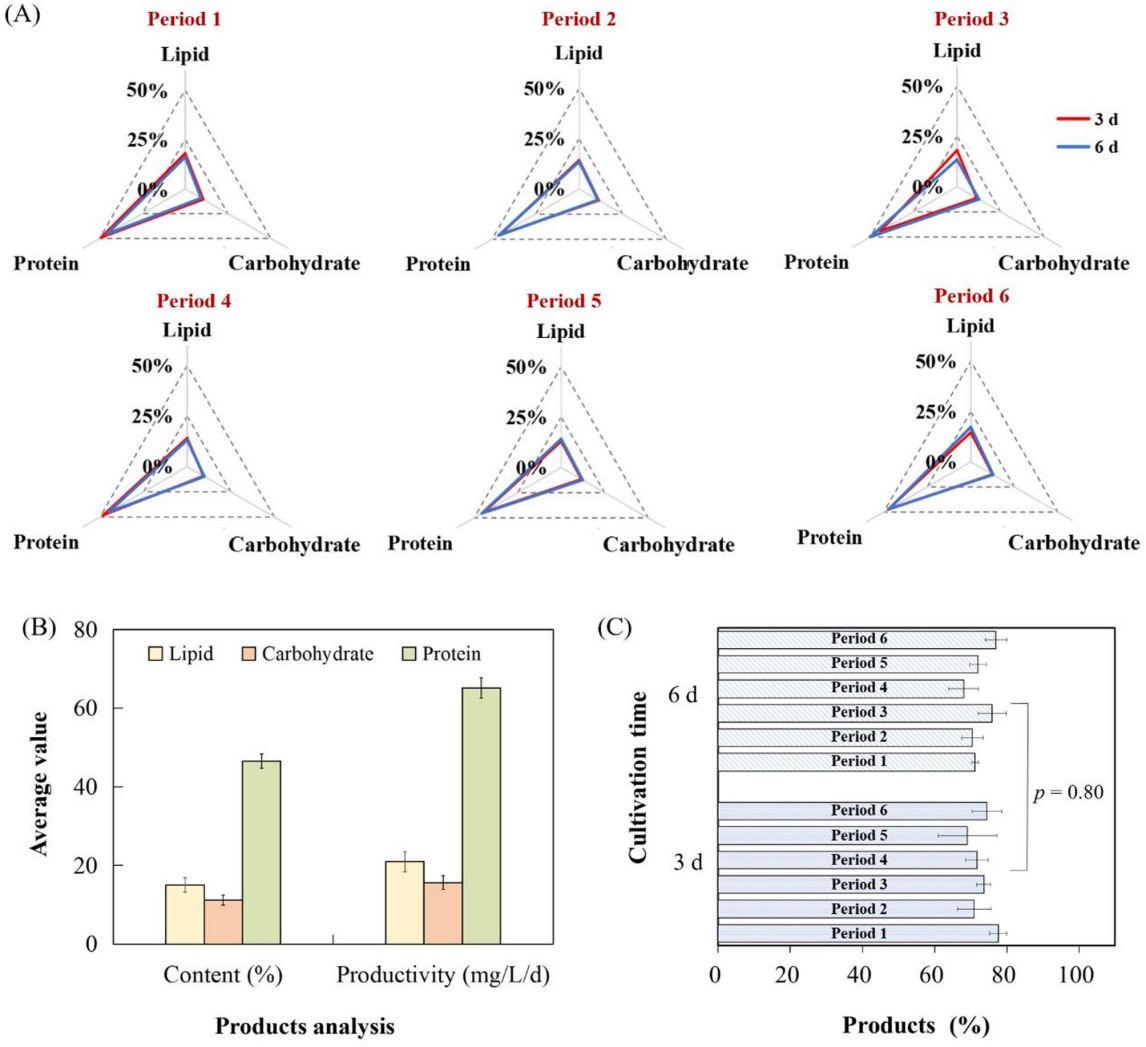
The main products of *Spirulina subsalsa* from the long–term cultivation. (**A**) Lipid, protein, and carbohydrate content within six periods; (**B**) average lipid, protein, and carbohydrate content and productivity of *Spirulina subsalsa*; and (**C**) the overall product content of *Spirulina subsalsa* powder harvested on the 3^rd^ and 6^th^ days within six periods

Algal products were determined as depicted in Fig. 6. Among those products, protein content was maintained at 44.17–49.92%, which was higher than the lipid content (13.05–18.12%) and the carbohydrate content (9.27–12.78%). This was because *Spirulina subsalsa* prioritizes rapid growth over energy storage, allocating carbon and nitrogen resources primarily to protein synthesis rather than lipid and carbohydrate. Additionally, MSGW, which is rich in ammonium nitrogen, further promotes protein synthesis over carbon sequestration. Given the high protein content of *Spirulina subsalsa*, there is considerable potential for the future development of protein-based products. The mean productivities of lipid and carbohydrate of *Spirulina subsalsa* were respectively 20.94 and 15.21 mg/L/d, which were each less than a third of protein productivity. Hence, this *Spirulina subsalsa* has the potential to be widely used as a high-quality protein source in the future.

According to Fig. 6, the average contents of lipid, carbohydrate and protein were respectively 14.95%, 10.87% and 46.50% through the six periods. According to Andrade et al. (2019), the protein content of *Spirulina* sp. LEB 18 decreased significantly in reused Zarrouk medium. However, in this study, the composition of the algal product remained relatively consistent during the first three days and after three days. This indicated that the cultivation method in this study can stably produce algal powder with a consistent biomass composition. Total content of the three products accounted for 72.68% of the dry mas, with no significant difference in overall content harvested on the 3^rd^ and 6^th^ days. To sum up, it is feasible to stably produce *Spirulina subsalsa* powder with high protein content on a pilot scale.

## Economic analysis and carbon emission reduction of producing algal powder

**Fig. 7:**
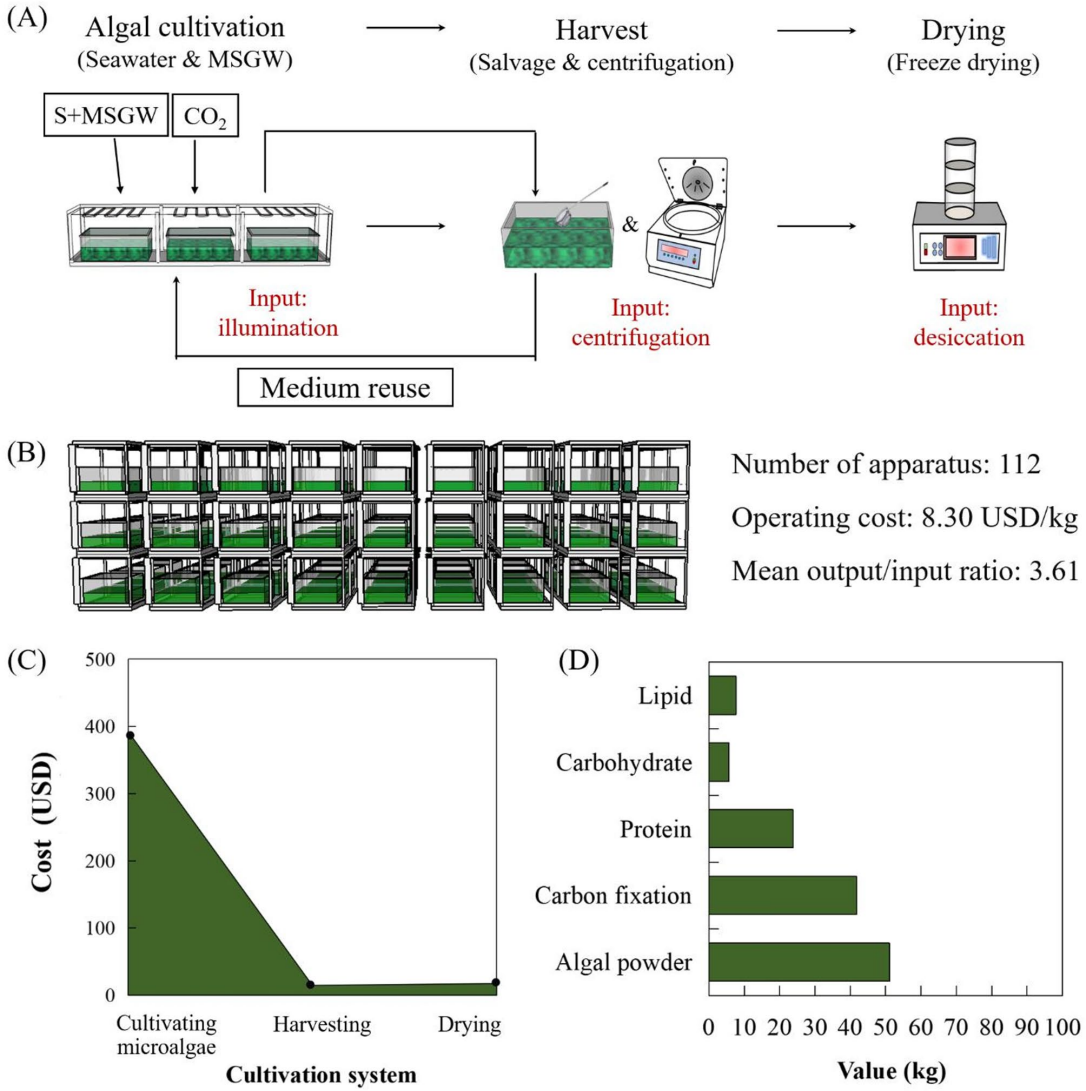
Economic analysis of *Spirulina subsalsa* for 1 m^3^ scale over a one-year period. (**A**) The inputs and outputs in the different stages of algal production. (**B**) Diagram of 1 m^3^ scale for culturing *Spirulina subsalsa*. (**C**) Cost analysis of *Spirulina subsalsa* production in the different stages. (**D**) The value of carbon fixation, produced algal powder and products

Based on pilot-scale data, economic analysis of algal production was performed for the 1 m^3^ medium over a one-year period (Fig. 7). For operating inputs, the relevant processes include cultivation, harvesting, and drying. During the cultivation process, the expenditure was primarily attributable to the culture medium and electricity. The medium for cultivating *Spirulina subsalsa* was composed of natural seawater and wastewater, thus the nutrient cost was not a primary consideration. As stated in the literature by Yuan et al. (2018), the cost of traditional chemical culture media is USD0.08 per litre. In this study, our cultivation method has saved USD80 in nutrition costs compared with the traditional chemical culture media. Since *Spirulina subsalsa* thrived without aeration, there’s no need for expenditures related to microalgal mixing. Regarding the algal illumination, the consumed electricity was 4905.6 kWh for operating 112 lamps, each with a rating of 0.010 kW and working for 12 h/d. With the electricity price of 0.08 USD/kWh, the expenditure for cultivation would be 392.45 USD. During the process of harvesting and drying, the primary cost factor was electricity required for centrifugation and freeze-drying. The *Spirulina subsalsa* was primarily harvested using a scoop net, followed by a series of centrifugations, and then dried by a freeze dryer. The consumed electricity was 399 kWh and the operating costs for harvesting and drying would amount to 31.92 USD.

From the foregoing, the operating expenditure would be 424.37 USD in total. Based on the commercial price of *Spirulina subsalsa* powder (10–50 USD/kg), the output of microalgal biomass would amount to 511–2555 USD. For a median value of approximately 30 USD/kg *Spirulina subsalsa* powder, output/input ratio would be 3.61, representing a positive return. The production cost of algal powder was 8.30 USD/kg dry biomass, which is lower than 10.84 USD/kg dry biomass for culturing *Arthrospira* sp. and 13.2–17.3 €/kg dry biomass for culturing *Spirulina* sp. (Chaiklahan et al. 2018; Kingsley et al. 2023). Moreover, the total electricity consumption was 103.81 kWh/kg dry biomass. According to Fig. 7 (C), the electricity cost during the cultivation stage was the crucial factor in microalgal production. When continuously scaling up the algal production system, more energy-efficient illumination systems for large-scale cultivation would be utilized to save electric power resources. In terms of harvesting and drying, larger centrifuges and freeze-dryers are able to process more algae per unit time, which have a lower unit power consumption. This directly reduces the cost per unit of dry biomass production, enhancing economic feasibility. However, the location selection should be in areas where seawater and wastewater are readily available. Moreover, it is crucial to ensure that there is a sufficient market for the algal products to avoid over-supply and price drops.

The value of carbon fixation, produced algal powder and products was shown in Fig. 7 (D). During the microalgae production process, 52.20 kg of CO_2_ was sequestered, thereby realizing a reduction in carbon footprint. The total dry mass of microalgae attained 51.10 kg, which contains 23.76 kg protein, demonstrating significant potential for the development of protein-based products. In conclusion, the production of *Spirulina subsalsa* demonstrates considerable potential and offers significant advantages in terms of environmental sustainability, economic benefits and nutritional value.

### The vision of the future

#### The applications of algal powder

Given that the protein content of *Spirulina subsalsa* ranges from 44.17 to 49.92%, the peptide composed of amino acids could be produced in large quantities for incorporation into foodstuffs, thus improving its function and nutritional value. In a study conducted by Silva et al. (2021), the addition of 2% bioactive peptides derived from *Spirulina* sp. LEB 18 resulted in a notable enhancement of antioxidant activity in extruded snacks. For protein-based products, the development of bioactive peptides has potential. Furthermore, a diverse range of other ingredients, including vitamins, polysaccharides, carotene, γ-linolenic acid, and phenolic compounds, also demonstrate considerable potential in health foods, especially with the enhancement of refining and extraction techniques.

The polysaccharide derived from Spirulina is composed of xylose, glucose and rhamnose, which play a certain role in anti-radiation, anti-fatigue, lowering blood glucose and lipids, strengthening immune function and so on (Silva et al. 2018; Rajasekar et al. 2019). Through the model of oxidative stress, polysaccharide derived from *Spirulina subsalsa* has been demonstrated to mitigate the effects of oxidative stress on cellular damage and applied in skin care products (data not published). Based on these advantages, the vision is put forward whereby the polysaccharides are extracted first and then the proteins are reused. Polysaccharides from this *Spirulina subsalsa* were extracted by ultrasonic extraction, hot water extraction, microwave-assisted extraction or enzymatic methods (Patel et al. 2022). After extraction, the protein content in the extracted algal residue will be increased which can be further applied in all walks of life.

#### Integrated utilization and intelligent system in microalgal production

Future research will focus on optimizing microalgal production strategies through three key approaches. Firstly, the aquaculture wastewater composed of MSGW and seawater was unreasonable utilization. Salt-containing wastewater from various industries is regarded as a potential salt resource and previous studies showed that 98% of salts were recovered in high-salt coal chemical wastewater, with 94% pure Na_2_SO_4_ and 96% pure NaCl (Liu et al. 2021; Chen et al. 2021). The treatment cost was 2.3 USD m^− 3^, and “zero discharge” of wastewater was economically realized through evaporation crystallization technology. Next, intelligent and informative production models are crucial for commercial microalgal production (Lim et al. 2022), enabling precise monitoring of key parameters and utilizing machine learning for predictive and automated optimization. The systems for industrialized cultivation of Spirulina chiefly comprise open raceway ponds and closed photobioreactors, respectively accounting for 83% and 17% (Araújo et al. 2021). For industrialized Spirulina production, it is essential to explore an intelligent cultivation strategy, especially in open cultivation systems. Further research is required on intelligent cultivation for *Spirulina subsalsa* based on distinctive characteristics. In addition, food industry effluents (brewery wastewater, dairy wastewater, beverage processing wastewater) should be further investigated to develop optimal wastewater-seawater hybrid systems for *Spirulina subsalsa* cultivation. And the life cycle assessment will be incorporated into the research framework to propose targeted optimizations by identifing high-impact stages.

## Conclusion

For this vertically migrating *Spirulina subsalsa*, it was suitable in non-aerated conditions and a wide-shallow reactor. Subsequently optimal depth and surface area were 4.5 cm and 2000 cm^2^ in a single apparatus. In a 162 L pilot-scale cultivation system, *Spirulina subsalsa* grew well with a culture medium of seawater plus MSGW. Across six periods, the average dry mass of microalgae on a pilot scale was 0.84 g/L. Nutrient assimilation indicated that average N-AYC and P-AYC of *Spirulina subsalsa* was 56.33 and 4.97 mg/g. The main metabolite composition of this *Spirulina subsalsa* was extremely stable, among which the mean protein content and productivity were 46.50% and 65.11 mg/L/d. Economic analysis showed that produced algal powder was 8.30 USD/kg. Moreover, there is potential to develop multiple products from *Spirulina subsalsa* in the future.

## Supplementary Information

Below is the link to the electronic supplementary material.

Supplementary Material 1

Supplementary Material 2

## Data Availability

Data will be made available on request.

## Abbreviations

MSGW: Monosodium glutamate wastewater
SP: Spirulina medium
TN: The total nitrogen
TP: The total phosphorus
NH_3_-N: Ammonium nitrogen
DOC: Dissolved organic carbon
DM: Dry mass
EEM: Excitation–emission matrix
N-ARC, P-ARC or C-ARC: Average removal coefficients of nitrogen, phosphorous and carbon
CER: Carbon emission reduction
