# Supplementary material for: Cultivation strategy optimization and pilot-scale production of Spirulina subsalsa grown in seawater and monosodium glutamate wastewater
Source: Bioresour Bioprocess. 2025 Jul 31;12(1):83. doi: 10.1186/s40643-025-00926-0 (PMC12314176; doi:10.1186/s40643-025-00926-0)
Supplement: Supplementary file 1 — Supplementary Material 1 [file 40643_2025_926_MOESM1_ESM.docx]

**Cultivation strategy optimization and pilot-scale production of *Spirulina subsalsa* grown in seawater and monosodium glutamate wastewater**

Mingyan Liu^1^, Liqun Jiang^2^, Ze Yu^2^, Meng Ma^1^, Huiying Chen^1^, Haiyan Pei ^1, 2, 3, 4^[[1]](#footnote-1)^*^

*^1^* *School of Environmental Science and Engineering, Shandong University, Qingdao, 266237, China*

*^2^ Department of Environmental Science and Engineering, Fudan University, Shanghai, 200433, China*

*^3^ Shandong Provincial Engineering Center on Environmental Science and Technology, Jinan, 250061, China*

### *^4^ Institute of Eco-Chongming (IEC), Shanghai, 202162, China.*

###
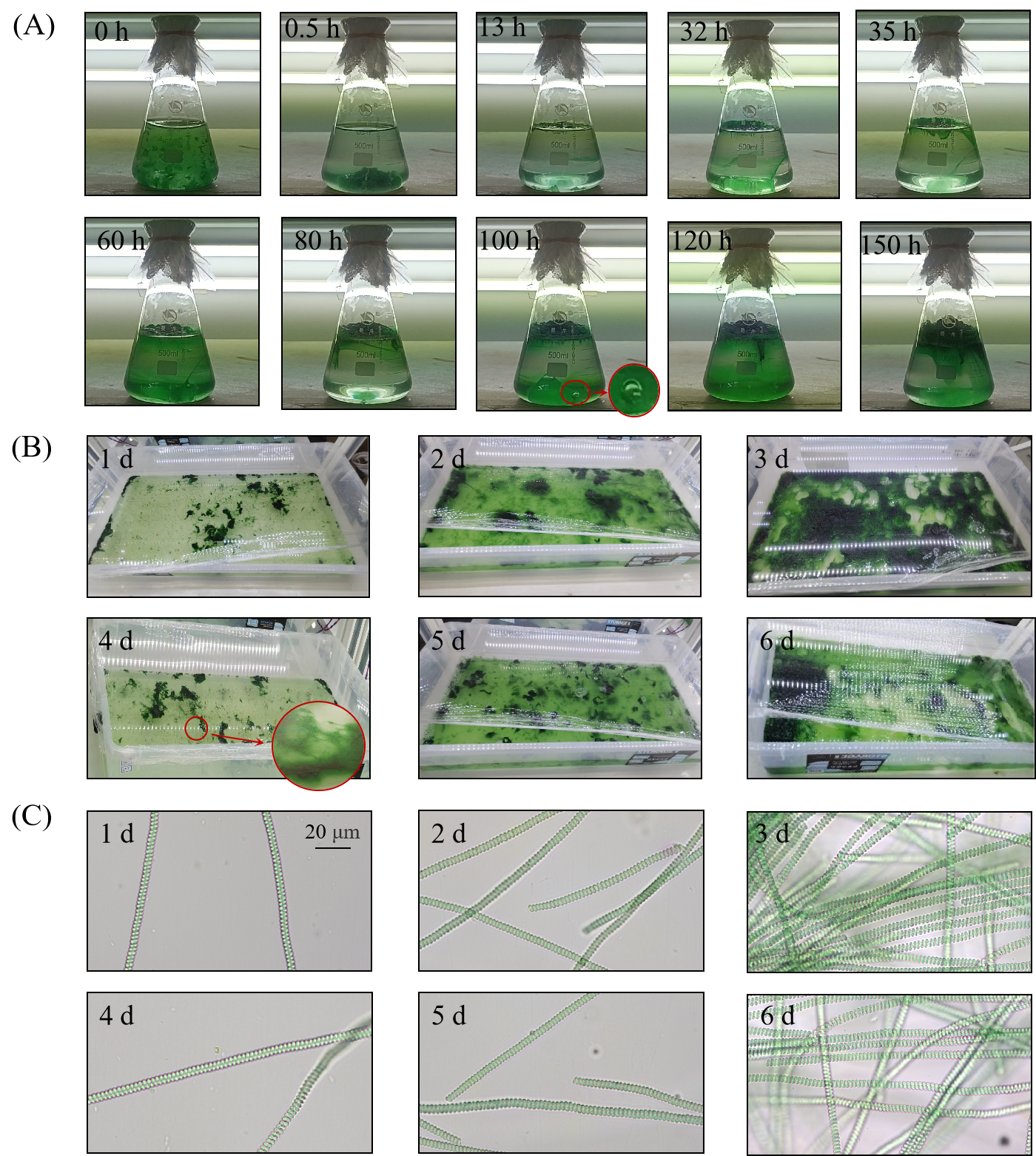


**Figure S1.** Photographs of the *Spirulina subsalsa* growth*.* (A) Photographs of *Spirulina subsalsa* with the trait of vertical migration. (B) Photographs of *Spirulina subsalsa* in a single incubator with optimal depth and area. (C) Micrographs of *Spirulina subsalsa* grown in the S+MSGW medium with optimal depth and area. The cultivation period was six days, harvesting on the third and sixth day.


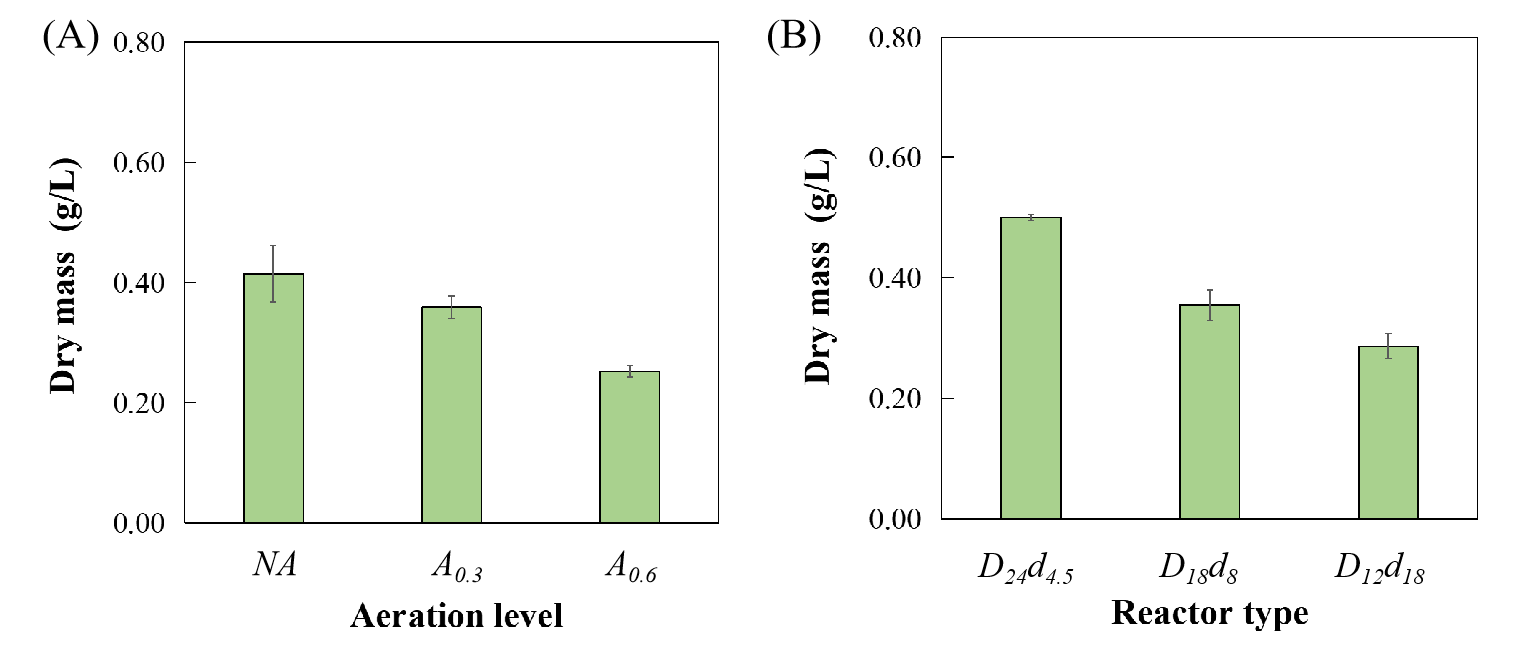


**Figure S2.** The cultivation mode optimization of *Spirulina subsalsa*. (A) The microalgal dry mass in non-aeration (*NA*)*,* aeration with the aeration rates of 0.3 (*A_0.3_*) and 0.6 (*A_0.6_*) L/min (B) The microalgal dry mass with diverse diameters (*D*, cm) and depths (*d*, cm; *D_24_d_4.5_*, *D_18_d_8_* and *D_12_d_18_*) in non-aeration conditions.

1. * Corresponding author:

   E-mail address: haiyanhup@126.com [↑](#footnote-ref-1)
