# Supplementary figures and images for: Cultivation strategy optimization and pilot-scale production of Spirulina subsalsa grown in seawater and monosodium glutamate wastewater
Source: Bioresour Bioprocess. 2025 Jul 31;12(1):83. doi: 10.1186/s40643-025-00926-0 (PMC12314176; doi:10.1186/s40643-025-00926-0)

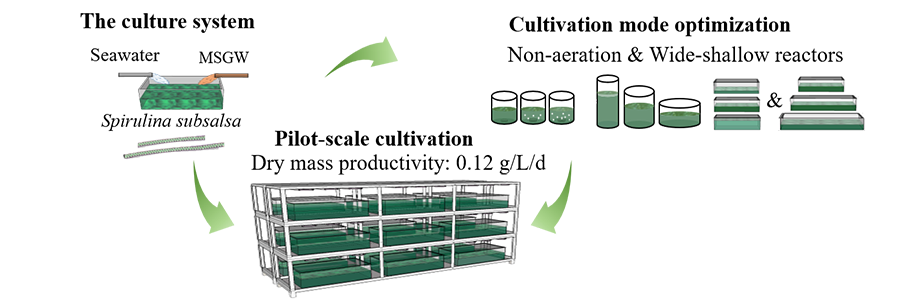

Supplement: Supplementary file 2 — Supplementary Material 2 [file 40643_2025_926_MOESM2_ESM.png]
